# Surgical treatment vs. conservative treatment in intravenous bisphosphonate-related osteonecrosis of the jaws. Systematic review

**DOI:** 10.4317/jced.53504

**Published:** 2017-02-01

**Authors:** Aida Comas-Calonge, Rui Figueiredo, Cosme Gay-Escoda

**Affiliations:** 1 DDS. Faculty of Dentistry – University of Barcelona; 2 DDS, MS, PhD. Master degree program in Oral Surgery and Implantology. Associate professor of Oral Surgery and Professor of the Master degree program of Oral Surgery and Implantology. Faculty of Dentistry – University of Barcelona. Researcher of the IDIBELL institute; 3 MD, DDS, MS, PhD. Chairman and Professor of Oral and Maxillofacial Surgery. Faculty of Dentistry – University of Barcelona. Coordinating investigator of the IDIBELL institute. Head of the Oral and Maxillofacial Surgery Department, Teknon Medical Center. Barcelona, Spain

## Abstract

**Aims:**

To determine the success rates of the surgical and non-surgical treatments in the management of bisphosphonate-related osteonecrosis of the jaws (BRONJ).

**Material and Methods:**

A systematic review of the literature was made. A PubMed Medline database search was performed in order to include clinical studies published in English,between2004 and 2014 with the following key-words: “BRONJ AND treatment” and “NOT osteoporosis”. The following data was gathered: authors, title, year of publication, aim of study, level of evidence, sample size, treatment performed, treatment outcomes and follow-up. Studies including more than 20 patients with at least 6 months of follow-up, and that specify the different treatment approaches and their outcomes were included. Systematic reviews were excluded.All studies were classified according to the SORT criteria (Strength of Recommendation Taxonomy).

**Results:**

The initial electronic search yielded 169 papers, and 13 studies were added after a manual search (total of 182 studies). After analysing the title and abstract and removing duplicates, 31 full-texts were obtained. A total of 12 papers were finally included. Two were classified as level 3 evidence and 9 as level 2. The quality of the selected studies and the risk of bias were also reported.

**Conclusions:**

Surgical treatments like sequestrectomy, surgical debridement and bone osteotomies provide successful treatment outcomes, with success rates ranging from 58 to 100%. Controlled randomized clinical trials with larger samples and longer follow-up are needed to support these findings.

** Key words:**BRONJ, treatment.

## Introduction

In 2003, Marx reported 36 cases of necrotic bone exposed in the jaws associated with the long-term use of bisphosphonates (1). Since then, a high number of cases of bisphosphonate-related osteonecrosis of the jaws (BRONJ) have been documented. Most BRONJ are associated with the intravenous bisphosphonates administration (IVBP), although some cases have also been described after oral intake of these agents (2).

In 2009, the American Association of Oral and Maxillofacial Surgery (AAOMS) published a staging system in order to classify each case of BRONJ according to its signs and symptoms; and to propose different treatment approach (3).

Several therapeutic strategies have been recommended in the literature according to the severity of this complication, ranging from strictly conservative to aggressive surgical approaches (4). The treatment of BRONJ is still under debate, and most reports show different outcomes (5).

In the latest stage-dependent recommendations of the AAOMS, Ruggiero *et al.* (3) proposed a conservative regime with antibiotics, antibacterial mouthrinses, and pain control in stage 0 and stage I. When the patient is classified in stage II, a superficial debridement with removal of the bone sequestrum is recommended to relieve soft tissue irritation and finally, in stage III, surgical debridement with partial or total bone resection of the jaws should be considered. However, these recommendations are not widely followed. In fact, many papers suggest different approaches with varying success rates, depending on the characteristics of the sample. For this reason, a systematic review of the available literature was made in order to assess which treatment has a higher success rate in patients diagnosed with BRONJ.

## Material and Methods

A literature search was performed using the MedlinePubMed database in April 2014 with the following key-words: “BRONJ AND treatment” and “NOT osteoporosis”. Papers published in the last 10 years were analyzed and studies that addressed only oral bisphosphonates patients were excluded. When samples were composed by both IV and oral bisphosphonates, a separate analysis was made, retrieving only the IVBP data.

The following inclusion criteria were applied: papers in English, studies in humans, samples with more than 20 patients, trials that described the applied treatment and outcome. Systematic reviews and studies with a post-treatment follow-up of less than 6 months were excluded.

Two independent researchers decided the inclusion and/or exclusion criteria of the studies included in the present systematic review.

The following variables of each article were recorded: number of cases, age, gender, primary diagnosis, IVBP drug, duration of the IVBP treatment, BRONJ location, staging (according to Ruggeiro *et al.* (3)), and applied therapy. Also, treatment outcomes and follow-up time were assessed. Papers that did not record all these variables were excluded.

The treatment was considered successful when the patient improved the stage of the disease or when there was absence of bone exposure with proper healing, and the patient remained asymptomatic without any clinical signs of infection.

This systematic review followed-up the PRISMA guidelines (Preferred Reporting Items for Systematic Reviews and Meta-Analyses) (6) and all papers were classified according to their scientific evidence level using the SORT criteria ( Table 1).

**Table 1:**
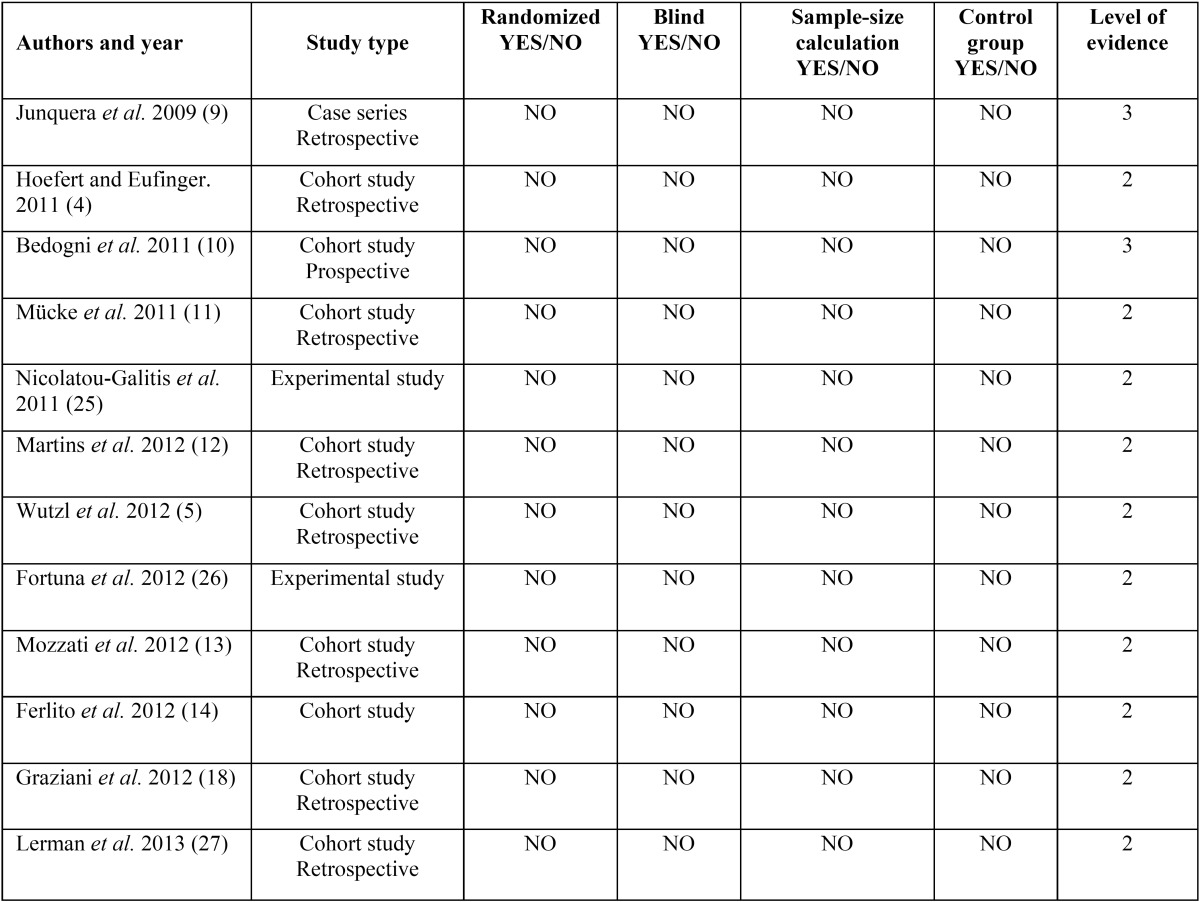
Scientific evidence level of the reports included in this systematic review.

## Results

**Figure 1:**
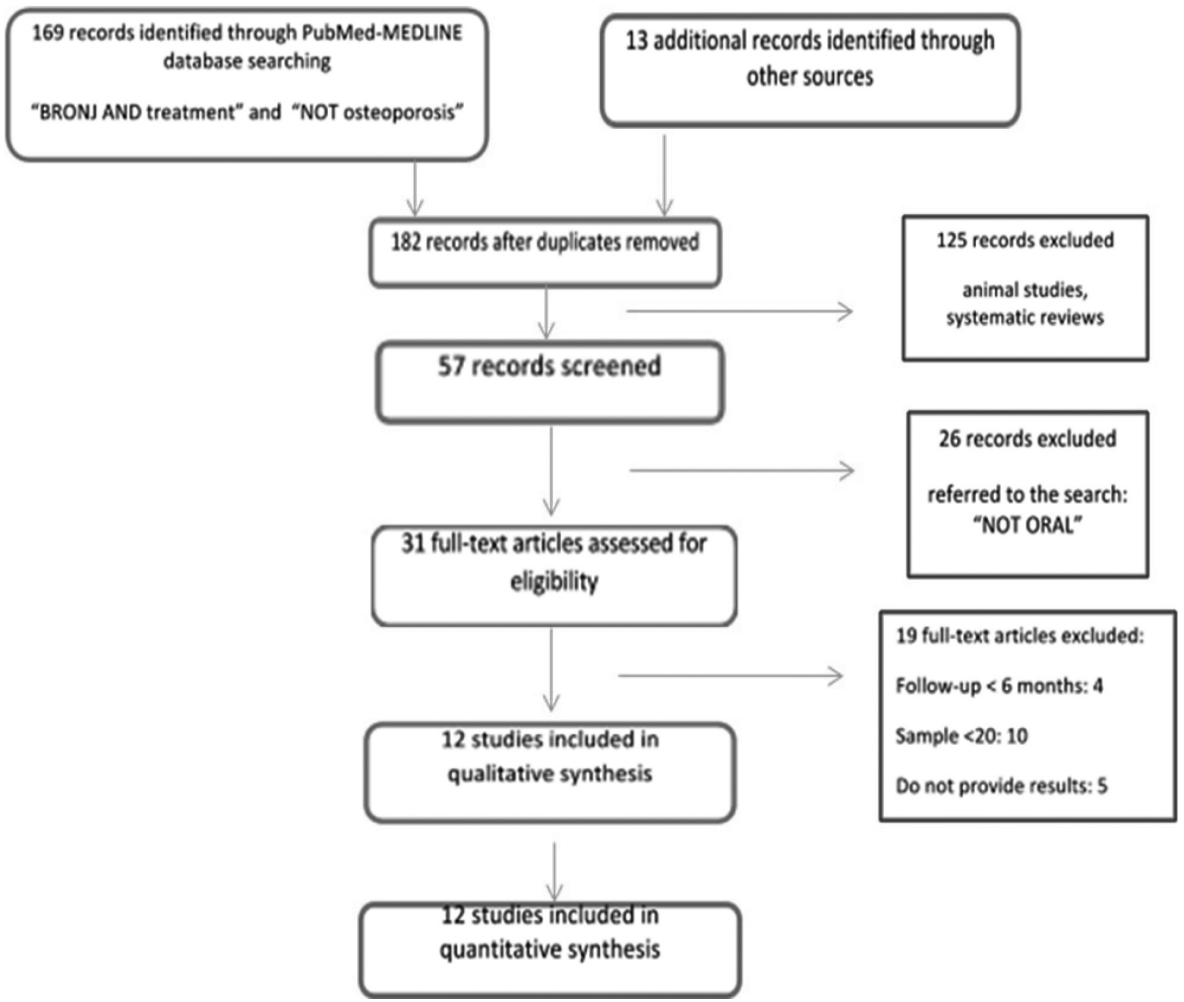
Flow-chart diagram search strategy.

The initial electronic search using the terms “BRONJ” AND “treatment” NOT “osteoporosis” yielded 169 papers (Fig. 1), and 13 studies were added after a manual search (total of 182 studies). After an initial evaluation, the authors decided to include the search term “NOT oral”, resulting in 57 relevant papers. After analysing the title and abstract and removing duplicates, 31 full-texts were obtained. A total of 12 papers complying with the inclusion/exclusion criteria were finally included. The level of evidence of the papers included in this systematic review according to the SORT classification can be seen in  Table 1.

Finally, the quantitative synthesis of the 12 papers of this systematic review is presented in  Table 2. The success rate of the surgical management of BRONJ ranges from 58% to 100%, while the pharmacological approach, based on antibacterial agents (antibiotics and antiseptic mouthrinses), has worse results (range: 33% to 100%).

**Table 2:**
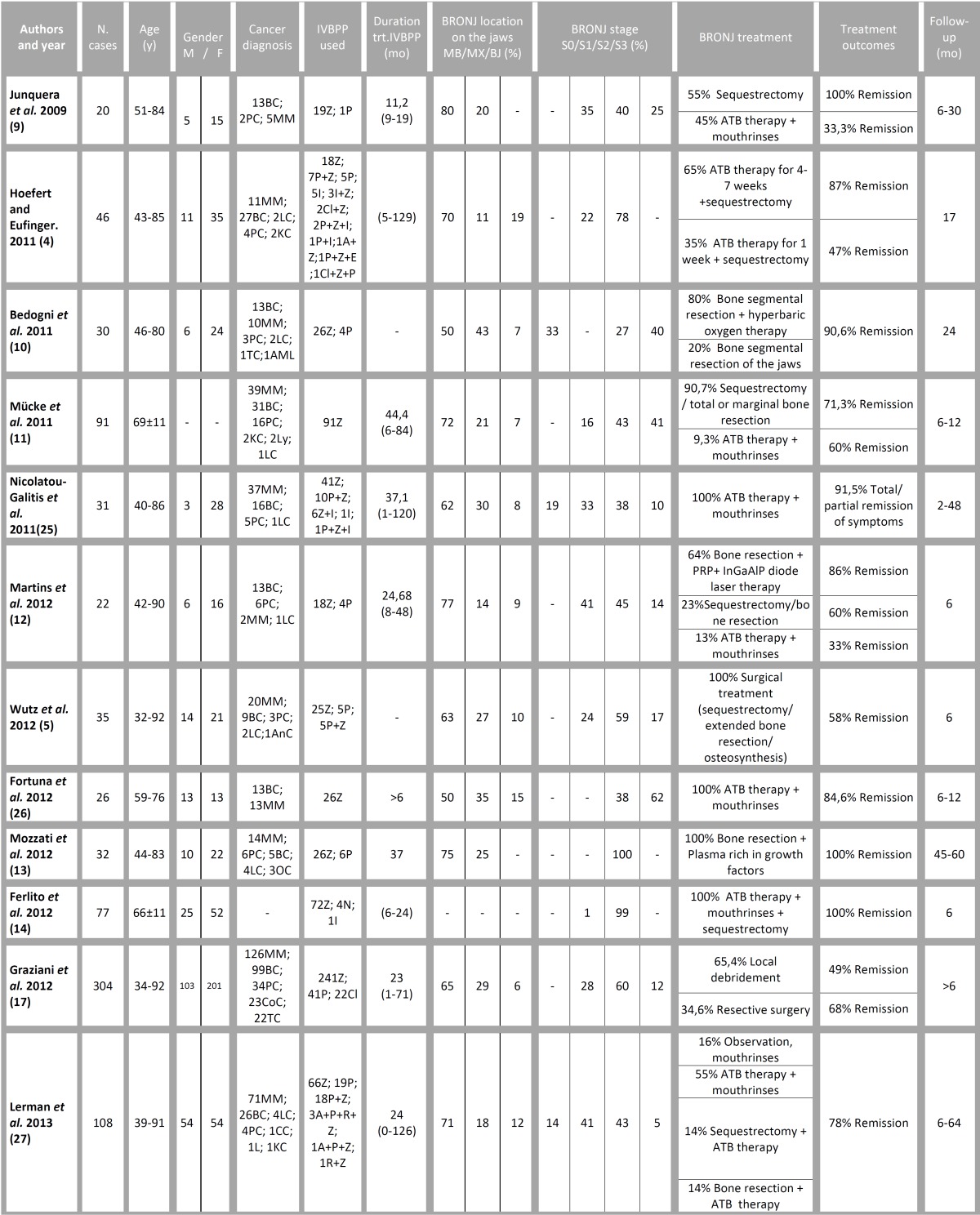
Studies selected according to the inclusion criteria. Features of the cases of each study, BRONJ treatment and outcomes.

## Discussion

The lack of standardized success criteria and treatment protocols is one of the main limitations of this review. Furthermore, the use of several types of surgical therapies (sequestrectomy and bone resection among others) and its association with several antibiotics and antiseptics also makes difficult to draw conclusions on which is the best treatment for BRONJ.

One of the advantages of using a more conservative surgical approach like sequestrectomy is that a better healing should be expected since the periosteum and unaffected bone are conserved (7). On the other hand, a correct bone resection with a tension-free closure of soft tissues allows achievable and predictable results. In a recent review, the authors observed a 85-100% of success rate in wound healing without complications after surgery (8).

Most authors (6,9-14) achieved a good result with a surgical treatment based either in local debridement and sequestrectomy or in bone resection. Aggressive therapies with segmental resection of the affected bone with inclusion of the periosteal layer combined with hyperbaric oxygen therapy (HBO) also seem effective (10). Bedogni *et al.* (10), reconstructed these cases with titanium plates and local soft and hard-tissue flaps and achieved a complete remission in 80% of patients after 24 months of follow-up. HBO therapy also showed positive effects in 62,5% of the cases of another study (15) but it should not be considered as an individual treatment modality for BRONJ at this time (16,17).

In the retrospective study of Hoefert and Eufinger (4), cases were divided into two groups treated with the same surgical technique but with two different antibiotic regimens. After 17 months of follow-up, the cases treated with a long-term preoperative antibiotic therapy had better results (complete healing in 87% of the cases in contrast to 47% with a short-term regimen). Furthermore, the authors state that the combination of extensive surgical bone resection with antimicrobial mouth rinses and prolonged antibiotic therapy may lead to complete healing (4). Ferlito *et al.* (14) also emphasised the importance of the use of suitable antibiotics, anti-inflammatories, analgesics and mouthrinses to minimise pain and infection before the formation of a bony sequestrum.

Graziani *et al.* (18) and Carlson and Basile (19) propose a more aggressive management, based in bone resections, to treat BRONJ patients. Regardless the stage of the disease, areas of necrotic bone that are a constant source of soft tissue irritation should be removed in order to allow a proper healing (3,17).

Most patients under bisphosphonate therapy are usually also receiving other therapeutic agents, such as corticosteroids, statins, and other chemotherapeutic agents, all of which could have a significant effect on the incidence of BRONJ (7). This statement has been supported on the recent update position paper of the AAOMS published in October 2014 (17).

Tension-free closure of the wound and an adequate bone resection are key factors for the treatment prognosis. Although it is extremely difficult to quantify the amount of bone that should be removed, bleeding is considered a sign of healthy bone, although the reliability of this sign is a controversial subject (11,20,21).

Laser might be an interesting alternative to conventional bone removal devices. Vescovi *et al.* (22) proposed the use of Erbium-doped Yttrium Aluminium Garnet (Er:YAG) laser, achieving a complete remission of the signs and symptoms in all the patients of their trial. The same authors also established the use of low-level laser therapy (LLLT) in BRONJ treatment using a Neodymium-doped Yttrium Aluminium Garnet (Nd:YAG) laser.

A combined treatment protocol consisting in pharmacological therapy, surgical treatment with platelet rich plasma (PRP) and laser phototherapy (LPT) using Indium-Gallium-Alluminium-Phosphide (InGaAlP) diode laser, has shown positive results (64% success rate) in the management of BRONJ patients (12). Some authors (13,23) use PRP or plasma rich in growth factors (PRGF) due to its capacity to improve soft tissue healing.

The routine use of antibiotics (both preoperative and postoperative), and of antiseptic mouthrinses generally with chlorhexidine digluconate 0.2% is recommended by the vast majority of authors. Bacteria play an important role in the physiopathology of BRONJ and seem to be directly involved in the development of necrotic lesions and in the inhibition of epithelial regeneration over the exposed bone (24). Once extended BRONJ lesions are present, systemic antibiotics are not able to reach the affected area due to the lack of vascularisation. In fact, Junquera *et al.* (9) and Mücke *et al.* (11) concluded that BRONJ patients with advanced stages would not improve when treated only with antibiotics since progression of the lesions will occur.

However, a conservative approach based in the administration of antibiotics, antiseptics, analgesics, antifungals and fluorides, seems to be particular effective in the initial stages of the disease (stages 0 and I) (25,26). Both Nicolau-Galitis *et al.* (25) and Fortuna *et al.* (26) obtained, respectively, a 91,5% and a 84,6% success rates with this approach. Other authors (27,28) also used minimally invasive protocols with good results. Indeed, Montebugnoli *et al.* (28), concluded that there was no significant difference in the amount of necrotic bone between the samples of cases treated with surgery and the ones treated only with antibiotics.

Considering the results of the publications included in our review, the success rates of BRONJ surgical treatment vary between 58-100%. The different surgical techniques, the sample characteristics (type of IVBP; duration of BP therapy; presence of additional risk factors and location of the lesions), the lack of well-defined success criteria and the additional treatments performed (antibiotics, antiseptics and LLLT) justify this variation of outcomes. Our results seem to support the results of a recent systematic review (29), which concluded that extensive surgery and laser seem to provide the best healing results. On the other hand, the level of scientific evidence provided by the studies published to date (type 2 and 3), does not allow drawing any sound conclusions. Indeed, there is a great need to perform well design controlled randomized clinical trials in order to increase the degree of recommendation. In addition, in order to facilitate future research on this topic, it would be especially interesting to unify the success criteria.

## Conclusions

- The absence of level 1 scientific evidence studies does not allow recommending any treatment approach for BRONJ patients. There is clearly a need to performed large sample controlled randomized clinical trials with an adequate follow-up.

- A wide range of treatment protocols has been published with varying results. The lack of standardized success criteria makes comparisons between treatments very difficult.

- In light presentations of BRONJ (classified as degree 0 and 1), a conservative approach based in prescription of antibacterial agents seems to be the most adequate approach. On the other hand, a surgical treatment based in sequestrectomy, surgical debridement and/or bone removal provides successful treatment outcomes, with a 58-100% success rate.

- All treatments described have a low grade of recommendation according to the SORT criteria.
